# Establishing a Protocol to Characterize Sludge from Oil and Water Lines

**DOI:** 10.1021/acsomega.5c04136

**Published:** 2025-10-27

**Authors:** Gustavo G. Celestino, Júlia Veiga Nunes, Elizabete F. Lucas

**Affiliations:** † Programa de Engenharia Metalúrgica e de Materiais/COPPE/LADPOL, 28125 Universidade Federal do Rio de Janeiro, Av. Horácio Macedo, 2030, bloco F, Rio de Janeiro 21941598, Rio de Janeiro, Brazil; ‡ Instituto de Macromoléculas/LMCP, Universidade Federal do Rio de Janeiro, Rua Moniz Aragão, 360, bloco 8G/CT2, Rio de Janeiro 21941594, Rio de Janeiro, Brazil

## Abstract

During well drilling, production, processing, transportation, and refining of the crude oil, it is common to observe sludge formation, which can contain several kinds of compounds, such as organic compounds of the oil and inorganic compounds generated during oil and/or water production. There are many procedures to prevent organic and inorganic deposition; however, to apply suitable procedures, it is necessary to identify the cause of the sludge formation. This can be achieved through a detailed sludge characterization. This study presents a sequence of techniques to characterize different kinds of industrial sludges and proposes the causes of the formation of each of them. The techniques used involved thermogravimetric analysis, successive solvent extractions, microcalorimetry, sulfide test, X-ray fluorescence, X-ray diffraction, and scanning electron microscopy. The sequence of the analyses was presented considering the amount of sample available. Four industrial sludge samples were used, two from an oil production line and two from a water production line. The protocol established to characterize the sludge was shown to be useful to identify the cause of the deposition. The main causes detected in the samples analyzed were asphaltene deposition, inorganic deposition, and inorganic compounds formed by hydrogen sulfide and corrosion products.

## Graphical abstract

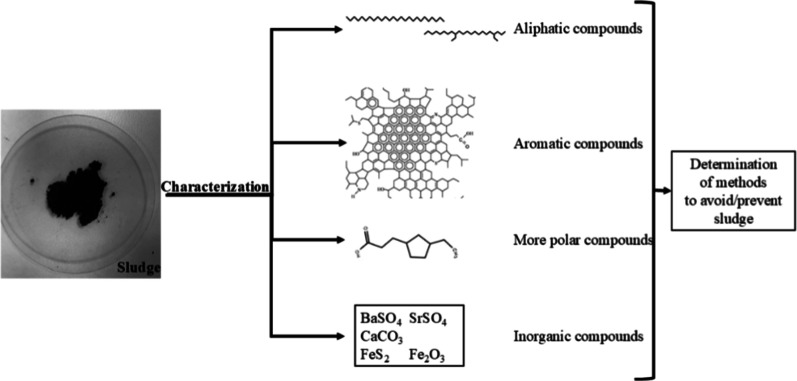

## 1 Introduction

During well drilling, production, processing, transportation, and refining of the crude oil, it is common to observe sludge formation. Flow assurance problems can be related to organic and inorganic deposition, emulsion, chemical incompatibility, corrosion, and sludge formation. Sludges are usually generated in the oil and/or water production lines and can be constituted for several kinds of compounds, such as organic (waxes, asphaltenes, and salts of naphthenic acids) and inorganic compounds (carbonates, sulfates, and corrosion products), alone or combined with each other.^1–17^

Sludges are considered complex emulsions presenting a general composition of 30–80% of oil, 30–50% of water, and 5–46% of solids.^18,19^ It is estimated that, in refineries, sludge formation represents around 40% of operating costs and 50% of investment costs.^17^ Nowadays, some companies use treatments to recycle oily materials and/or eliminate sludge using techniques such as incineration, pyrolysis, photocatalysis, ultrasonic treatment, and biodegradation, and most published articles focus on this topic.^18,20–27^

Although these treatments are being used to recover oil from sludges, applying methods to prevent sludge formation is quite interesting for the petroleum industry. However, to prevent the formation of such deposits, it is necessary to characterize the sludges to obtain information about the percentages of the different classes of compounds.^4,8,12,13,28,29^

There are few studies in the literature aiming for the characterization of sludges in terms of the quantification of the percentage of oil in the sludge. Some of them use extractions, followed by specific techniques to determine the oil content. For example, Hu et al.^30^ applied the extraction method using chloroform, dichloromethane, or ethyl acetate at different sludge/solvent proportions and determined the best condition to recover the highest amount of oil. Similarly, Wang et al.^31^ performed extractions using toluene, benzene, or petroleum ether to determine the percentage of oil in sludge, by gravimetry. The greatest efficiency in extracting the oily phase (25.8%) was achieved using hot toluene. Lima et al.^32^ separated organic and inorganic fractions using a Soxhlet extraction with chloroform, determining their percentages. Then, they determined the saturated, aromatic, resins, and asphaltenes (SARA) composition and characterized the inorganic phase by X-ray fluorescence. Jasmine and Mukherji^33^ quantified iron, calcium, and magnesium in the sludge, using scanning electron microscopy-energy-dispersive X-ray spectroscopy directly in the sludge. They also used column chromatography, with the sequential addition of different solvents, to separate fractions from the maltenes extracted from the sludge. It allowed them to determine the percentage of each fraction in the sludge.

The procedures found in the literature present purposes that are a little different among them, and no systematic protocol to characterize the chemical composition to evidence the causes of the sludge formation was found. Sludge characterization must be carried out using techniques that allow the determination of organic and inorganic characteristics as well as the chemical characteristics of each class of these compounds. Therefore, the aim of this study was to establish a consistent protocol to characterize sludges using not only successive Soxhlet extractions, with solvents with different polarities, but also other characterization techniques, such as thermogravimetric analysis (TGA), Karl Fisher titration (KFT), microcalorimetry (μDSC), X-ray diffraction (XRD), scanning electron microscopy (SEM), X-ray fluorescence (XRF), and sulfide test. In this study, 12 sludge samples were obtained in the oil industry from oil and water production lines.

## 2 Experimental Section

### 2.1 Materials

Twelve sludge samples from a Brazilian oil field were donated by Equinor Brasil: five from the oil production line (OPL01 to OPL05) and seven from the water production line (WPL01 to WPL07). A Karl Fischer reagent was acquired from Honeywell Fluka, Rio de Janeiro, Brazil. Undecane (99%) was purchased from TCI America. Toluene, *n*-heptane, and dichloromethane were purchased from Isofar, Duque de Caxias, Brazil. The lead acetate (analytical grade), sulfuric acid (95%), and wax (melting point between 56 and 58 °C) were supplied by Sigma-Aldrich, São Paulo, Brazil. Asphaltene 7CI was extracted from the crude oil.

### 2.2 Procedures

The strategy used to develop the protocol was to start with the extractions because extractions are the most used in the literature. Successive extractions using solvents with different polarities associated with gravimetric analyses could give us the percentages of different fractions and enough material to be analyzed by other techniques. It also included the determination of the water content because some sludges can present a large amount of water, being an emulsion well stabilized. Because the successive extraction procedure requires a relatively large amount of samples and is a time-consuming procedure, it was investigated the information given by thermogravimetric analyses performing the test under an inert and oxidative atmosphere in sequence using the same sample. Moreover, it can complement the information given by successive extractions.

To better identify the causes of sludge formation, microcalorimetry can be used to identify waxes, and to identify inorganic compounds in the residue obtained from the successive extractions, XRD, SEM, XRF, and sulfide tests were used.

#### 2.2.1 Successive Extractions Using Solvents with Different Polarities

Successive extractions were performed using the Soxhlet extractor and the following order of solvent addition: *n*-heptane, toluene, and dichloromethane. Detailing the procedure, ∼15 g of sludge was put into the cellulose cartridge and placed in a Soxhlet extractor containing 500 mL of *n*-heptane in the glass flask. The system was kept under reflux until the solvent became clear. The cartridge was reserved, and the solvent containing the extracted fraction was evaporated by vacuum distillation. The nondistillated fraction was placed in an oven at 70 °C until a constant weight. This mass is related to the percentage of aliphatic compounds. Using the reserved cartridge, the extraction was continued by adding 500 mL of toluene to the glass flask of the Soxhlet extractor. The same procedure described for the extraction with *n*-heptane was used to obtain the percentage of aromatic compounds. Finally, the cartridge was submitted to extraction with dichloromethane, following the same procedure to obtain the percentage of more polar compounds. The sample remaining in the cartridge, at the end of successive extractions, was dried until a constant weight, corresponding to the residue fraction.

#### 2.2.2 Karl Fischer Titration

The titration using a Karl Fischer reagent by biamperometry is classical to determine water content in samples.^34^ A procedure based on ASTM D4377 (ASTM D4377, 2018) was applied using a Karl Fischer 870 KF Titrino Plus Metrohm. The equipment was conditioned with the solvent (methanol: chloroform 4:1). Approximately 20 mg of the sludge sample was added to the titration vessel. The analysis was performed up to an electrometric end point using a Karl Fischer reagent. All analyses were done at least in duplicate because it must present a standard deviation of ≤1.0%.

#### 2.2.3 Thermogravimetric Analysis

Among other information, thermogravimetric analysis (TGA) allows one to obtain mass loss as a function of temperature. Organic compounds lose mass up to 800 °C, while inorganic compounds do not. The analysis requires 15 mg of the sample and is performed in about 2 h.^30,35^ The analyses were carried out using a thermogravimetric analyzer Q50 TA Instruments, using about 15 mg of the sludge. The sample was heated up from 30 to 800 °C at 10 °C/min under a N_2_ atmosphere. Then, the system was cooled to 40 °C at 10 °C/min. At this moment, N_2_ was replaced by synthetic air and the remaining sample was heated up again to 800 °C at 10 °C/min. The results were expressed as the percentage of mass loss versus temperature.

#### 2.2.4 Differential Scanning Microcalorimetry

Differential scanning microcalorimetry (μDSC) was used to verify the presence of an exothermic peak related to wax crystallization, using a Setaram μDSC VII-D3830 device. For that, 400 mg of the product extracted with *n*-heptane were added into the analysis cell. Undecane was used as the reference cell. Undecane is used because of its stability in the temperature range from −30 to 90 °C and its specific heat being similar to that of waxes. The analysis was conducted by heating up to 80 °C at a heat rate of 1 °C/min, keeping it for 10 min, cooling down to −10 °C at a cooling rate of 0.48 °C/min, keeping it for 10 min, and heating to 25 °C also at a heat rate of 1 °C/min. The wax appearance temperature (WAT) was defined as the crossover of the tangent line of the peak with the baseline of the curve obtained under cooling. The area integration of the peak provides the crystallization enthalpy because the equipment provides the heat flow (d*H*/d*t*) versus temperature (*T*). Each analysis takes about 5 h. The analyses were performed in duplicate, and the results were reproducible.

#### 2.2.5 X-ray Diffraction

X-ray diffraction (XRD) patterns of the residues were recorded in a Bruker D2-Phaser diffractometer, using Cu Kα radiation at 30 kV and 15 mA, over an angular range of 0–120° with a step size of 0.02 s^–1^, using 50 mg of the sample. This technique gives information about the crystallinity of the samples. The composition of the crystalline material can be speculated by using the equipment software library. The technique requires ∼50 mg of the sample and takes less than 2 h.

#### 2.2.6 Scanning Electron Microscopy

Scanning electron microscopy (SEM) of the residues was performed using 5 mg of the sample obtained after sequential extractions. The sample was placed on self-adhesive carbon tape and coated with a thin layer of gold. Images were obtained at 15 kV, using a Tescan Vega 3. SEM analysis requires ∼5 mg of sample and takes ∼30 min.

#### 2.2.7 XRF

The XRF analyses of the residues were conducted at Rigaku supermini 200 with a voltage of 50 k*V*, power of 200 W, and Pd anode. The samples were put in a film of prolene in the sample holder with a diameter of 44 mm, and the analyses were performed under vacuum. The sensitivity of the equipment comprises elements from fluorine to uranium. Elements with atomic numbers lower than the fluorescence are not detected. Elements were detected by F-PC and S-PC scintillation counters using a P10 gas. The technique requires ∼400 mg of the sample and takes less than 10 min.

#### 2.2.8 Sulfide Test

The sulfide test to analyze the residue was based on the reaction between hydrogen sulfide (H_2_S) and lead acetate­((CH_3_COO^–^)_2_Pb^2+^) as described in eqs 1,2, and 3.

1 $$1MS\left(s\right)+{2H}_{2}{\text{SO}}_{4}\left(aq\right)\to {1MSO}_{4}\left(aq\right)+{1H}_{2}S\left(g\right)$$

2 $${1Pb}^{2+}{\left({CH}_{3}{COO}^{-}\right)}_{2}\left(aq\right)+{1H}_{2}S\left(g\right)\to 1PbS\left(s\right)+{2CH}_{3}COOH$$

3 $${Pb}^{2+}+S^{-2}\to PbS,Kps={3\times 10}^{-28}$$

$$M={Fe}^{2+},{Ba}^{2+},{Sr}^{2+}$$

This test is a qualitative experiment, and the sulfide content cannot be quantified by using this technique. The laboratory procedure was carried out using 0.1 g of the residue, which was put in a test tube containing 5 mL of water. After this, 5 mL of concentrated sulfuric acid was added in the test tube, and filter paper moistened with a saturated solution of lead acetate was put over the test tube. The presence of sulfide provokes the color change of the filter paper from colorless to black. This procedure was applied to all residues obtained from successive extractions.

## 3 Results and Discussion

### 3.1 Successive Extractions Using Solvents with Different Polarities

The general procedure to characterize sludge is based on successive extractions using solvents with different polarities.^32,33,36^ Therefore, we start the analyses using successive extractions. Table 1 shows the results obtained for all of the sludge samples. The high content of aliphatic, aromatic, and more polar compounds could be related, for example, to the role of waxes, asphaltenes, and salts of naphthenic acid precipitation, on the sludge formation, respectively. The high content of the residue could be related to inorganic and/or water-soluble organic compounds being responsible for the sludge formation. The sludges generated in oil lines tend to present a higher content of aliphatic and/or aromatic compounds; while sludges formed in water lines tend to a present higher content of the residue. Figure 1 presents the aspect of the residues from OPL05, WPL06, and WPL07. In fact, this analysis gives a good profile of the sludge composition but it is still too generic. Moreover, it requires about 15 g of the sample and takes 4 days.

**Table 1:** Percentages of Aliphatic, Aromatic, More Polar Compounds, and Residue Obtained for all Sludge Samples by Successive Extractions with *n*-Heptane, Toluene, Dichloromethane, and Water Content

lines	samples	aliphatic (%)	aromatic (%)	more polar (%)	residues (%)	water content (%)
**oil production**	**OPL01**	42.95	42.77	10.97	0	2.28
	**OPL02**	11.71	53.80	5.26	0	5.47
	**OPL03**	39.94	43.06	3.73	0	9.17
	**OPL04**	41.07	37.62	0.03	0	10.36
	**OPL05**	11.00	10.70	2.70	67.94	1.57
**water production**	**WPL01**	6.36	14.31	5.42	43.69	13.80
	**WPL02**	1.64	5.69	0.92	75.83	4.67
	**WPL03**	4.00	9.50	0.90	54.32	28.00
	**WPL04**	4.64	1.70	0.15	85.98	5.45
	**WPL05**	1.93	1.91	2.45	47.90	39.93
	**WPL06**	4.69	6.42	1.15	68.70	3.93
	**WPL07**	34.29	9.22	0.70	50.10	3.11

**Figure 1:**
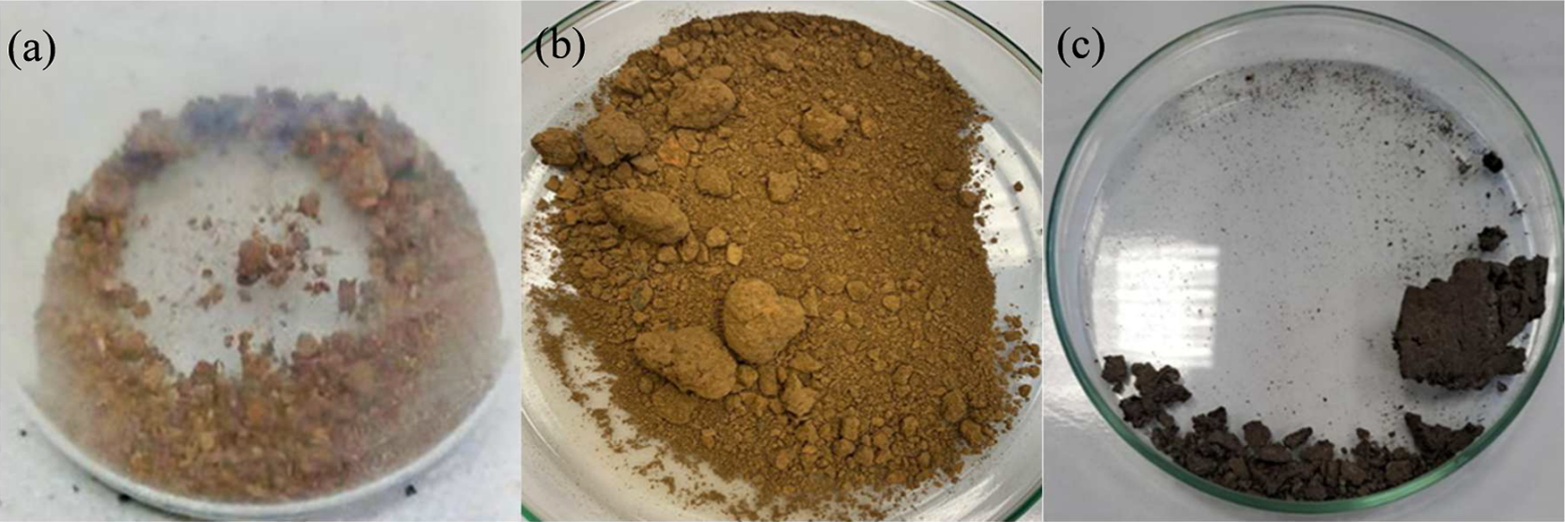
Aspect of the residue at the end of the successive extractions of the (a) OPL05, (b) WPL05, and (c) WPL07 sludge samples.

### 3.2 Water Content and Thermogravimetric Analysis

Generic information can also be provided by TGA and Karl Fischer Titration in about 2 h using, respectively, 15 and 20 mg of the sample. Because of this, we suggest starting the protocol to carry out these analyses, followed by successive extractions. Table 1 shows the water content for all of the samples. Although most samples present a low water content, some samples generated in water lines (WPL03 and WPL05) presented a relatively high percentage of water (respectively, 28 and 40%).

TGA can determine the percentages of the inorganic and organic compounds. The atmosphere can affect the mechanism of degradation. For example, in a range of the temperature between 30 and 800 °C, high molar mass aromatic compounds do not completely degrade under an inert atmosphere (N_2_), but they do under an oxidative atmosphere (containing O_2_), producing CO_2_ and H_2_O. On the other hand, inorganic compounds do not lose mass, under an inert atmosphere, at this range of temperature; and only a small variation of mass can be observed under an oxidative atmosphere because of oxidation. Therefore, the percentage of ashes obtained from the analysis under synthetic air can be related to the presence of inorganic compounds. The difference between the percentages of ashes obtained from the analyses under N_2_ and synthetic air can be related to the presence of high molar mass aromatic compounds, as follows:

Presence of inorganic compounds = percentage of ashes obtained from the analysis under an oxidative atmosphere (air).

Presence of high molar mass aromatic compounds = percentage of ashes obtained from the analysis under an inert atmosphere (N_2_)—percentage of ashes obtained from the analysis under an oxidative atmosphere (air).

Figure 2 shows the TGA analyses for OPL01 and OPL05. For comparison, the behavior of wax and asphaltene samples is also exhibited. Waxes completely degrade at around 400 °C under a N_2_ atmosphere. On the other hand, asphaltenes do not completely degrade under a N_2_ atmosphere but completely degrade at about 750 °C under synthetic air.

**Figure 2:**
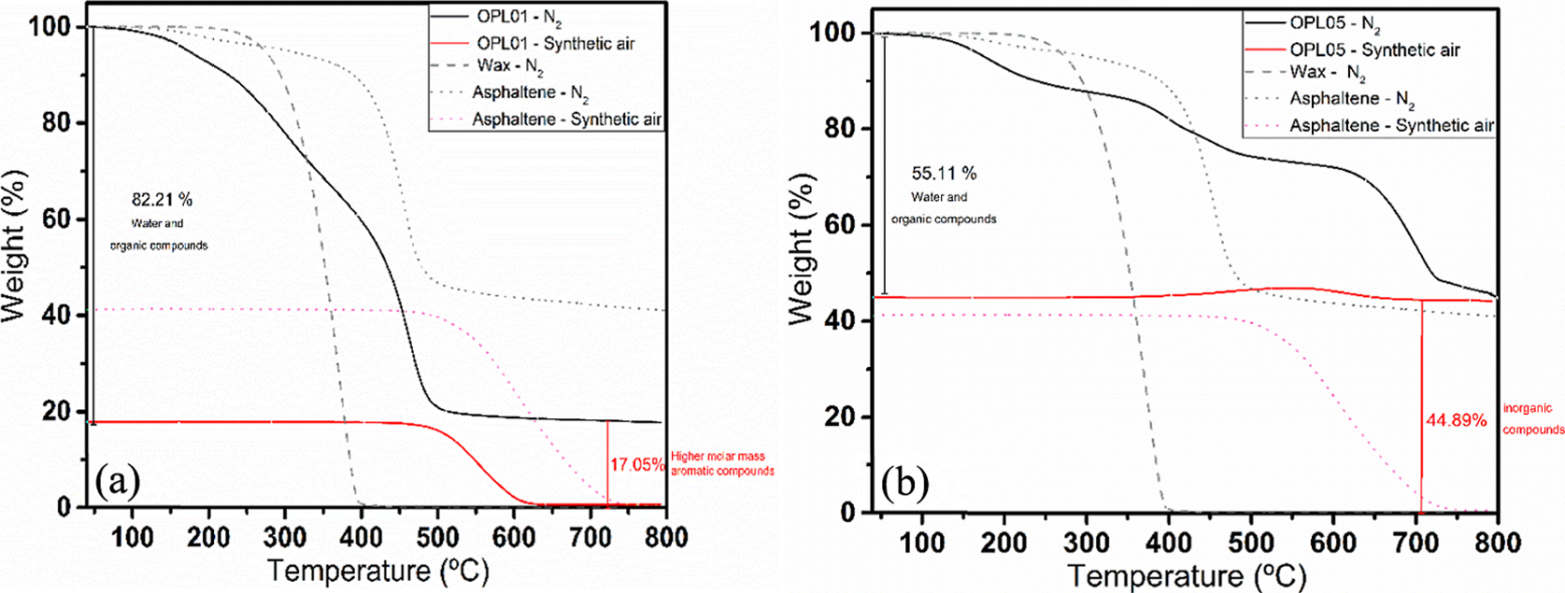
TGA curves of sludges (a) OPL01 and (b) OPL05. The analyses were done under N_2_ (black color) and synthetic air (red color).

Table 2 summarizes the TGA results obtained for all sludge samples. By comparing the results obtained by both methods, TGA and successive extractions (Table 1), we find they correlate well between them. No residue was obtained from the successive extractions for samples OPL01 to OPL04, and the presence of inorganic compounds was not detected by TGA. A higher percentages of aromatic compounds were found for OPL01, OPL02, OPL03, OPL04, OPL05, and WPL01, and the presence of asphaltenes was detected in the TGA for OPL01 to OPL04 and WPL01; no asphaltenes were detected in OPL05, indicating the presence of other lower molar mass aromatic compounds.

**Table 2:** Presence of Asphaltenes and Inorganic Compounds in all Sludge Samples Identified by TGA

sludge samples	ashes obtained under N_2_ (%) – ashes obtained under synthetic air (%)^a^	ashes obtained under a synthetic air atmosphere (%)^b^
	presence of asphaltene compounds	presence of inorganic compounds
**OPL01**	17.05	0.00
**OPL02**	10.23	0.00
**OPL03**	13.00	0.00
**OPL04**	11.00	0.00
**OPL05**	0.00	44.89
**WPL01**	10.00	31.00
**WPL02**	0.00	64.00
**WPL03**	0.00	52.00
**WPL04**	0.00	55.00
**WPL05**	0.00	36.00
**WPL06**	0.00	68.43
**WPL07**	0.00	38.00

a Aromatic compounds are degraded in oxidative atmosphere.

b Inorganic compounds are not degraded in any atmosphere.

From the successive extractions and water content (Table 1), and TGA analysis (Table 2), it is possible to strongly suggest that the formation of OPL02 was induced basically by asphaltene precipitation. It is hard to get conclusions about the other samples only by analyzing these results. Samples OPL01, OPL03, and OPL04 present similar percentages of aliphatic and aromatic compounds. Moreover, WPL07 also presents a significant amount of aliphatic compounds. To identify the possible role of wax precipitation on the formation of these sludges, microcalorimetry was included in the protocol to characterize the sludge.

### 3.3 Microcalorimetry

μDSC is a very sensitive analysis detecting the heat flow being absorbed or released by the sample during heating, cooling, or an isothermal program. It is quite useful to detect the wax appearance temperature (WAT) in crude oils during a cooling program, and the crystallization enthalpy variation (Δ*H*c).^4,5,12,37^

When a significant amount of the sample was taken from the extraction with *n*-heptane, the fraction was analyzed by μDSC to identify the presence of hydrocarbons with a high molar mass (waxes). Therefore, the aliphatic fractions obtained from the samples OPL01, OPL03, OPL04, and WPOL07 were analyzed by μDSC, as shown in Figure 3. The absence of an exothermic peak or the presence of a peak with very low enthalpy correspond, respectively, to the absence or the presence of a very small amount of waxes in the sludge. Therefore, the aliphatic fractions of samples OPL01, OPL03, and OPL04 do not present a significant amount of waxes, making it possible to suggest that the formation of these sludges was also induced by asphaltene precipitation.

**Figure 3:**
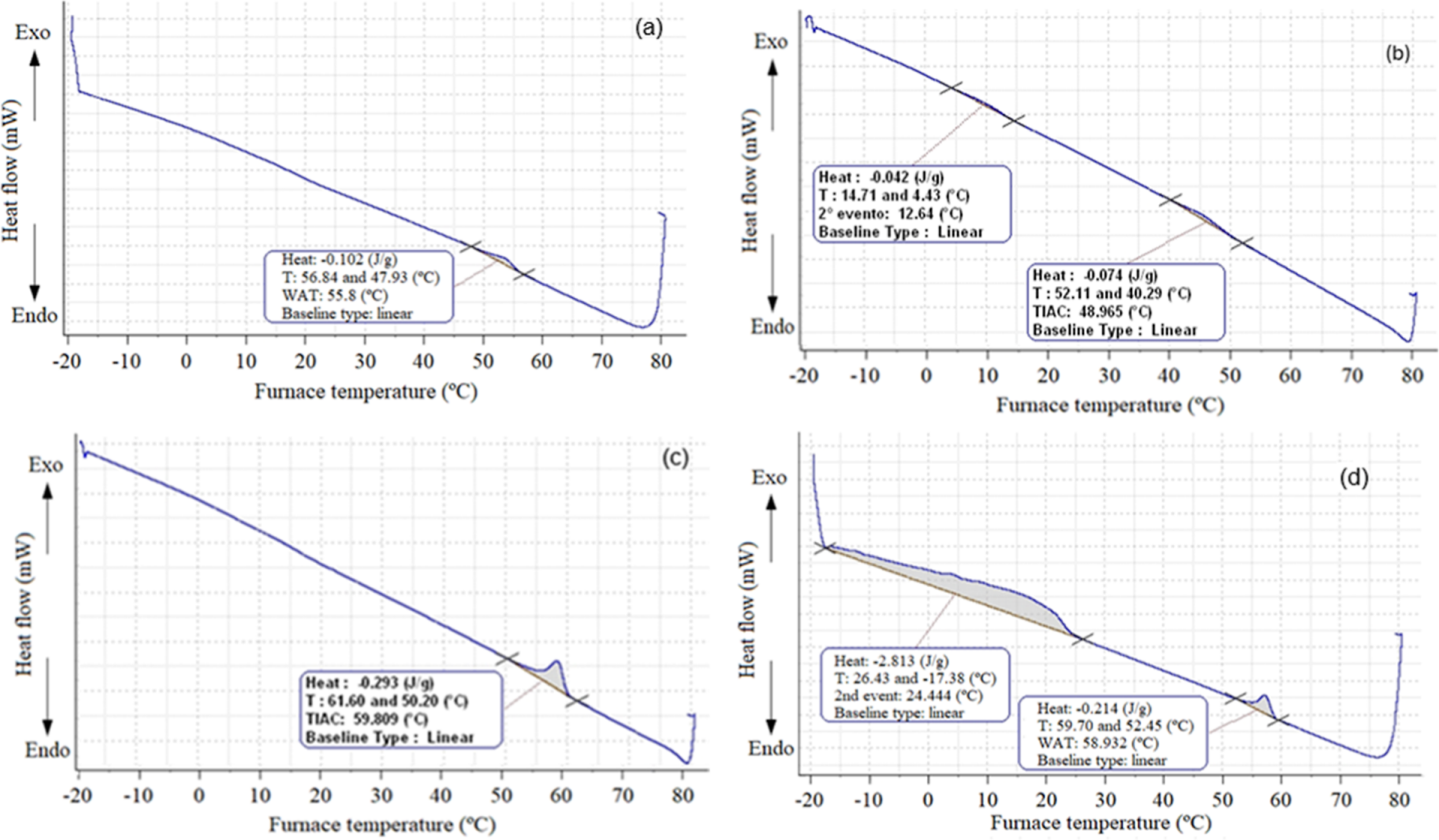
μDSC curves for the aliphatic fractions obtained from the samples: (a) OPL01, (b) OPL03, (c) OPL04, and (d) WPL07.

The μDSC curve of the aliphatic fraction obtained from WPL07 exhibited two exothermic peaks related to wax crystallization. However, this information alone cannot justify the waxes as the cause for sludge formation because it analyzed a concentrated fraction of aliphatic compounds. So, when a significant exothermic peak was detected, indicating the presence of waxes, the original sludge was also analyzed by μDSC (Figure 4) to evaluate the amount of wax in the sludge. It was calculated that about 20% of waxes were in WPL07. This percentage of wax in the sludge can be estimated by correlating Δ*H*_c_ values of the two samples (sludge and aliphatic fraction), as follows: Percentage of waxes in the sludge ≈ (Δ*H*_c_ of sludge/Δ*H*_c_ of aliphatic fraction) × 100. Although, it was possible to identify about 20% of waxes in WPL07, only 34% is related to aliphatic compounds; 50% is related to the residue (Table 1). Therefore, wax precipitation is not the only cause inducing sludge formation.

**Figure 4:**
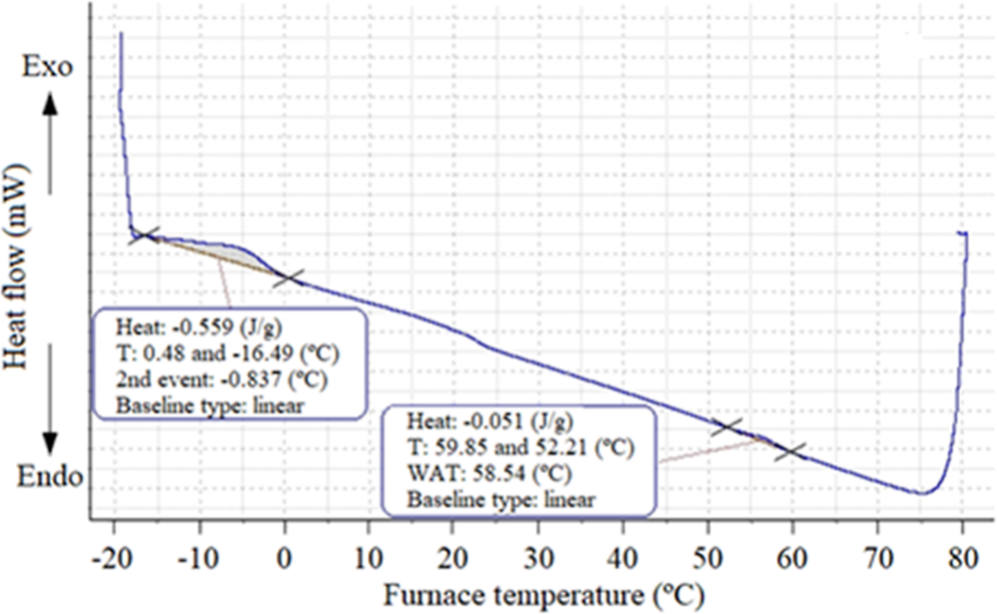
μDSC curve for the crude sludge WPL07.

In fact, all of the sludge generated in the water lines and one sludge formed in the oil line presented a large amount of residue after successive extractions. In these cases, it is not possible to infer about the cause for sludge formation only using successive extractions, water content, and TGA analysis. To investigate more deeply the compositions of these sludges, X-ray diffractometry (XRD), optional scanning electron microscopy (SEM), X-ray fluorescence (XRF), and sulfide testing were included in the protocol to determine the composition of materials insoluble in organic solvents.

### 3.4 X-ray Diffractometry

XDR is based on a phase relationship between two or more waves, which is essentially a scattering phenomenon. When X-ray beams are incident on a solid material, a fraction of the beam is scattered in all directions by the electrons that are associated with each atom or ion in the beam’s path. Most of the radiation scattered by one atom cancels out the radiation scattered by other atoms, but X-rays that fall on certain crystallographic planes, at specific angles, are diffracted instead of being canceled out. In this way, XRD is used to obtain information about the atomic and molecular structures of a crystal or to identify whether the sample does not present crystalline characteristics, that is, whether it is essentially amorphous. Different crystals present different responses, making it possible to obtain from the software library the better correspondence of the diffractogram with the chemical structure.^38^

Therefore, XRD is useful to inform about the morphology and to speculate about the crystal composition of the residues. Samples WPL01 to WPL05 presented an amorphous morphology. Figure 5 shows the XDR results for samples OPL05, WPL06, and WPL7, which exhibit crystallinity. According to the equipment library, the signals were attributed to the crystalline structure of calcium carbonate (CaCO_3_) for OPL05, and to calcium carbonate and cancrinite (Na_6_Ca_2_Al_6_Si_6_O_24_(CO_3_)_2_) for WPL06. Based on all results, the formation of OPL05 and WPL06 was induced by the precipitation of carbonate compounds. No specific identification was possible for crystals presented by WPL07 because of the overlapping signals of compounds based on carbonate and iron sulfide.

**Figure 5:**
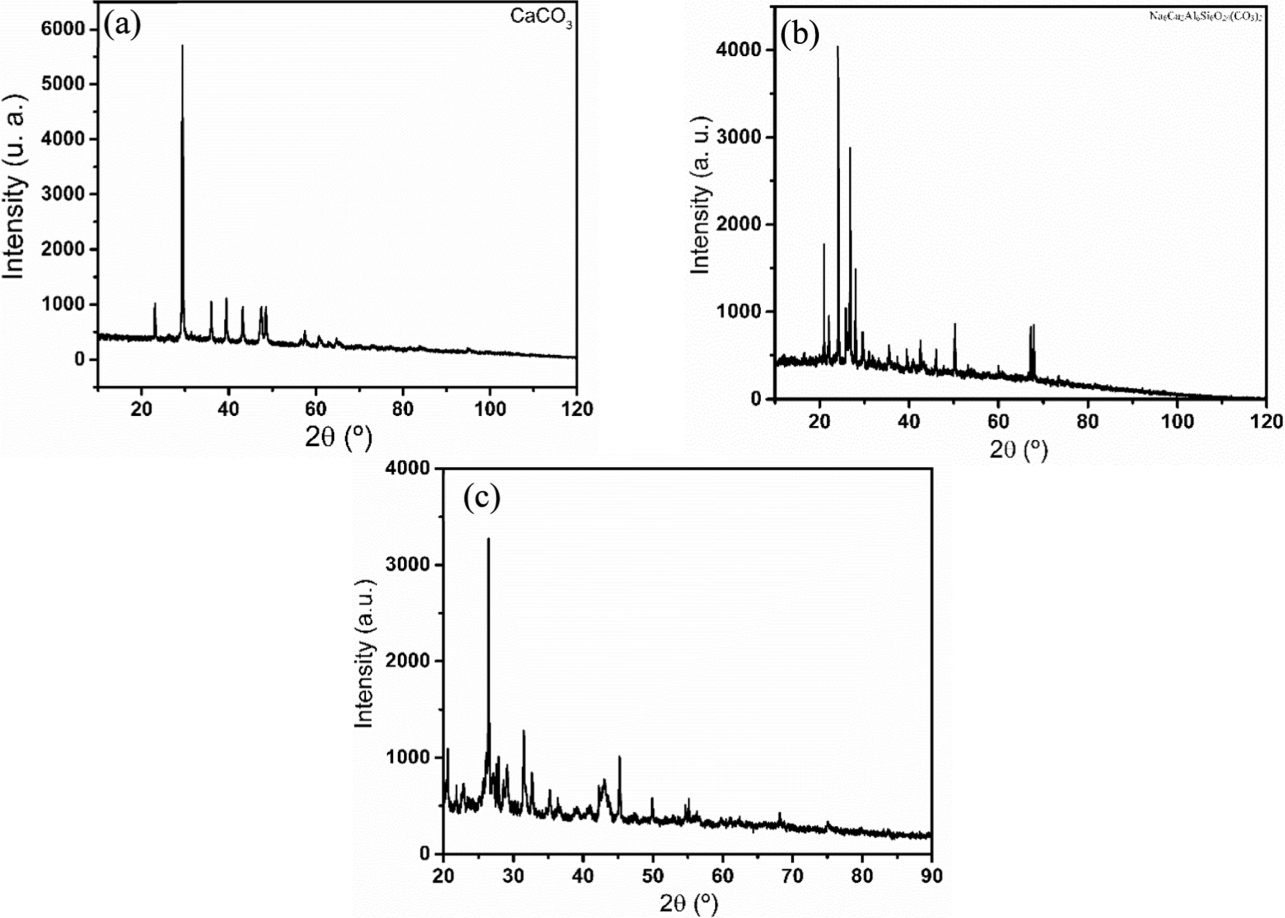
XRD of the residue extracted from (a) OPL05 (calcium carbonate), (b) WPL06 (cancrinite), and (c) WPL07 (iron or carbonate compounds) sludges after successive extractions.

#### 3.4.1 Scanning Electron Microscopy

SEM consists of using a small diameter electron beam to explore the surface of the sample, point by point, in successive lines and transmitting the signal from the detector to a cathode screen, the scan of which is perfectly synchronized with that of the incident beam. The beam interacts with the sample, producing electrons and photons that are collected by suitable detectors and converted into a video signal. This provides a photomicrography of the surface of the sample being able to observe its morphology.^39^

SEM can be applied to the residue to corroborate the results obtained by XRD. Optionally, SEM can be used to inform about the morphology instead of XRD when there is not enough residue available. As an example, Figure 6 shows the electron micrography of samples WPL03 (amorphous) and WPL06 (crystals of carbonates).

**Figure 6:**
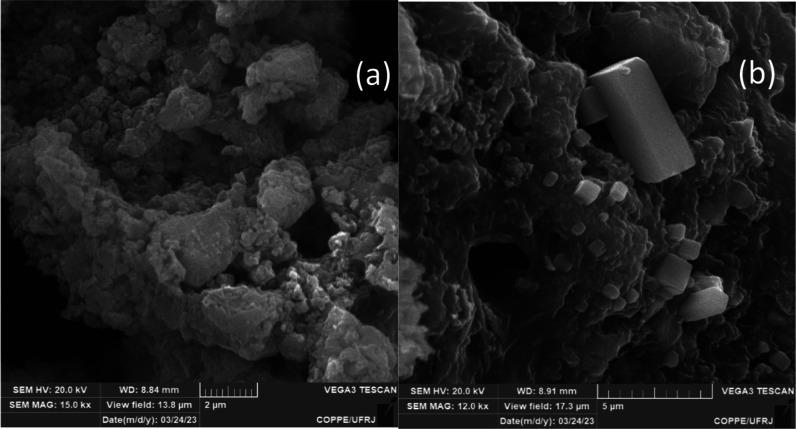
Scanning electron micrography of the residue obtained after successive extractions of (a) WPL03 (amorphous) and (b) WPL06 (crystalline) sludge samples.

### 3.5 X-ray Fluorescence

In the XRF technique, X-rays are focused on the sample to excite the electronic levels of the atoms. These atoms generate characteristic X-rays that are emitted from the sample. Such emissions are known as the fluorescence phenomenon and have specific wavelengths for each element. In this way, XRF allows the identification of the composition and concentration of elements present in a sample.^40^ Therefore, XRF allows us to quantify the metallic elements present in the residues obtained after successive extractions. Table 3 shows percentages of the main elements detected by XRF. All samples presented a significant amount of iron, which can be related to iron compounds from corrosion products. OPL05 and WPL07 presented the lowest content of iron. Based on all results obtained up to now, it can be strongly suggested that calcium carbonate is the main cause for sludge OPL05 formation and the precipitation of a mixture of inorganic salts is the main cause for sludge WPL07 formation, including some iron compounds and about 20% of waxes found in the previous analyses. XRF results make clear that iron compounds (34.2%) and some carbonated compounds are responsible for WPL06 formation. All other residues (WPL01 to WPL05) presented more than 60% of iron products, indicating that this is the main cause of these sludges’ formation.

**Table 3:** Percentages of the Main Elements Detected by XRF of the Residues Obtained after Successive Extractions of the Sludge Samples

residue of sludge	elements (%)						
	Fe	Si	Ca	Mg	Sr	Ba	S
**OPL05**	23.1	3.3	48.5	3.5	12.7	1.3	1.2
**WPL01**	83.5	5.2	1.5	0.4	1.2	1.1	2.5
**WPL02**	84.3	5.4	1.8	0.7	1.2	0.8	2.2
**WPL03**	80.1	6.5	1.4	0.6	1.1	1.2	2.2
**WPL04**	66.6	11.1	1.6	2.1	0.9	1.3	2.3
**WPL05**	63.2	10.1	1.7	1.5	0.8	1.0	2.6
**WPL06**	34.2	23.7	8.6	2.6	1.9	5.0	4.6
**WPL07**	22.9	12.2	2.7	1.0	8.4	24.6	7.2

### 3.6 Sulfide Test

Sulfide testing allows one to identify the presence or absence of sulfide compounds in the residue. The sequence of reactions involves the addition of strong acid (H_2_SO_4_) to the sludge residue. If the sample contains sulfide ion, the hydrogen sulfide gas (H_2_S) will be generated. This gas in contact with Pb^2+^ (lead acetate in the filter paper positioned on the glassware) generates a black color, which is related to PbS precipitation. Such precipitation is explained by the low-solubility product (*K*_sp_) on the order of 10^–30^ of PbS. Only sample WPL07 presented a black color in the filter paper, as shown in Figure 7. All other samples exhibited no color in this test. This indicates that at least some of the iron products in WPL07 were induced by sulfide compounds. This procedure requires ∼100 mg of the sample and takes less than 15 min.^41^

**Figure 7:**
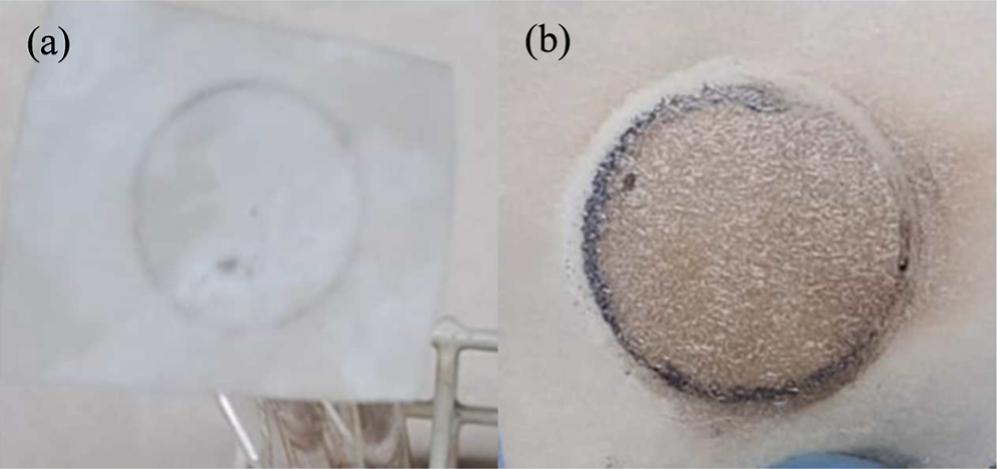
Filter paper after the sulfide test with residues of the (a) OPL05 and (b) WPL07 sludge samples. Black color in (b) corresponds to the precipitate of PbS, indicating the presence of sulfide compounds in the sludge residue.

Table 4 summarizes main characterization findings, reasons for sludge formation, and the respective recommendations to prevent such sludge formation for all sludges used in this study.

**Table 4:** Summary of Key Findings from Sludge Characterization, Reason for Sludge Formation, and Recommendations for Sludge Prevention

sludges	comments	conclusion	preventive recommendations
OPL01	large amount of aliphatic and aromatic compounds.	precipitation induced by aromatic compounds	injection of asphaltene stabilizer
	nonsignificant peak related to waxes		
OPL02	large amount of aromatic compounds (53.8%)	precipitation induced by aromatic compounds	injection of asphaltene stabilizer
OPL03	large amount of aliphatic and aromatic compounds.	precipitation induced by aromatic compounds	injection of asphaltene stabilizer
	nonsignificant peak related to waxes		
OPL04	large amount of aliphatic and aromatic compounds.	precipitation induced by aromatic compounds	injection of asphaltene stabilizer
	nonsignificant peak related to waxes		
OPL05	67.94% of residue containing a large amount of calcium (48.5%) and iron (23.07%) elements.	precipitation induced by carbonates and iron compounds	injection of scale inhibitor
	CaCO_3_ detected by XRD		
WPL01	43.69% of residue containing a large amount of iron (83.51%)	precipitation induced by iron compounds	injection of corrosion inhibitor
WPL02	75.83% of residue containing a large amount of iron (84.34%)	precipitation induced by iron compounds	injection of corrosion inhibitor
WPL03	54.32% of residue containing a large amount of iron (80.10%)	precipitation induced by iron compounds	injection of corrosion inhibitor
WPL04	85.98% of residue containing large amount of iron (66.63%)	precipitation induced by iron compounds	injection of corrosion inhibitor
WPL05	47.90% of residue containing large amount of iron (69.15%)	precipitation induced by iron compounds	injection of corrosion inhibitor
WPL06	68.7% of residue containing large amount of iron (34.18%), silicon (23.66%) and calcium (8.64%).	precipitation induced by carbonates and iron compounds	injection of corrosion inhibitor and scale inhibitor
	Na_6_Ca_2_Al_6_Si_6_O_24_(CO_3_)_2_ detected by XRD		
WPL07	large amount of aliphatic compounds, but nonsignificant peak related to waxes.	precipitation induced by iron compounds caused by sulfide ion	injection of corrosion inhibitor, scale inhibitor, and biocide
	50.10% of residue containing a large amount of iron (22.92%) and barium (24.63%). furthermore, it was also identified the sulfide-ion presence		

Based on the study using 12 sludge samples produced in the petroleum field (five from oil lines and seven from water lines), a protocol was established to characterize sludge. The protocol flowchart showing the suggested order of execution and the amounts required for each analysis is presented in Figure 8.

**Figure 8:**
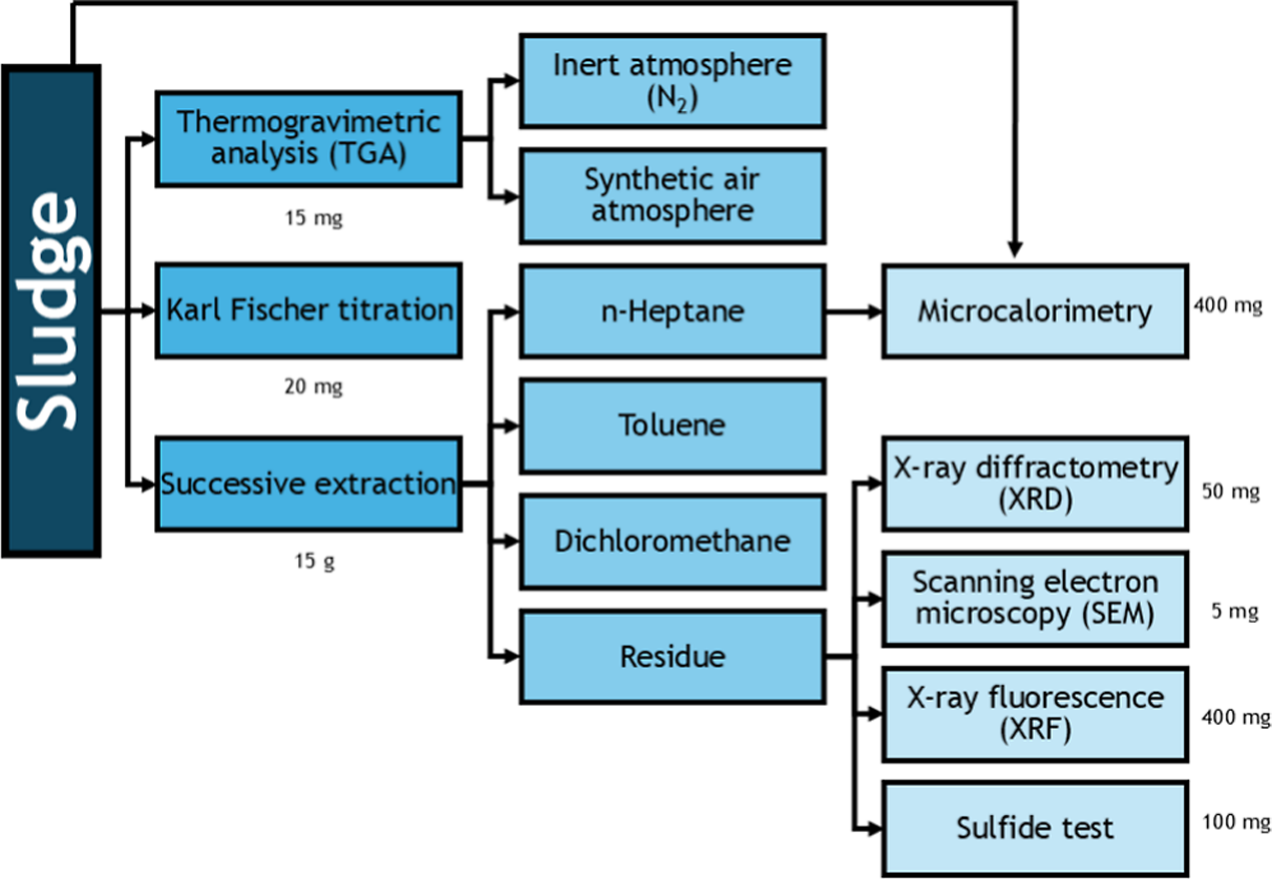
Flowchart of the protocol established for sludge characterization.

The developed protocol provides detailed information about sludge composition; however, it requires an advanced set of instrumentations. Considering the cost-benefit analysis, the techniques used can be recommended in the following order: (1) successive extraction, (2) sulfide test, (3) Karl Fischer titration, (4) TGA, (5) XRD, (6) XRF, (7), μDSC, and (8) SEM.

## 4 Conclusions

A consistent protocol to characterize sludge was established, using sludges obtained from the oil industry, making it possible to determine the reason for sludge formation. The formation of sludge withdrawals from the oil produced line were induced mainly by asphaltenes or calcium carbonate (CaCO_3_) precipitation. Concerning the sludge withdrawals from the water production line, their formation was mainly induced by corrosion products; or calcium carbonate and other carbonate compounds; or iron compounds (coming from corrosion products) and mixture of salts, besides a significant amount of waxes (∼20%). Thermogravimetric analysis has proven to be a simple, fast, and powerful technique to give important information about the sludge, for example, identifying the presence of asphaltenes and inorganic fraction. The successive extractions (using solvents presenting different polarities) combined with gravimetric analysis allowed us to quantify the percentages of aliphatic, aromatic, and more polar compounds, besides the residue. Moreover, it produced materials that could be analyzed by other techniques to better characterize the fractions. Microcalorimetry could identify how significant the waxes are in the sludge composition when a relatively large amount of the aliphatic fraction is isolated from the successive extractions. Finally, the residue could be well characterized by X-ray diffractometry, X-ray fluorescence, scanning electron microscopy, and sulfide test.

Although the protocol developed is capable of characterizing sludge generated in oil and water lines, future works should consider a methodology for identifying high molar mass water-soluble polymers in sludges to meet the needs of oil fields in which advanced oil recovery is applied.

## Nomeclature Section

ASTM: American Society for Testing and Materials
CH_3_COOH: acetic acid
CaCO_3_: calcium carbonate
Na_6_Ca_2_Al_6_Si_6_O_24_(CO_3_)_2_: cancrinite
CO_2_: carbon dioxide gas
Δ*H*c: crystallization enthalpy variation
μDSC: differential scanning microcalorimetry
H_2_S: hydrogen sulfite
KFT: Karl Fischer titration
(CH_3_COO^–^)_2_Pb^2+^: lead acetate
PbS: lead sulfite
N_2_: nitrogen gas
OPL: oil production line
O_2_: oxygen gas
SEM: scanning electron microscopy
*K*_ps_: solubility product constant
H_2_SO_4_: sulfuric acid
TGA: thermogravimetric analysis
H_2_O: water
WPL: water production line
WAT: wax appearance temperature
XRD: X-rays diffractometry
XRF: X-rays fluorescence.
